# Effects of Zn Fertilization on Hordein Transcripts at Early Developmental Stage of Barley Grain and Correlation with Increased Zn Concentration in the Mature Grain

**DOI:** 10.1371/journal.pone.0108546

**Published:** 2014-09-24

**Authors:** Mohammad Nasir Uddin, Agnieszka Kaczmarczyk, Eva Vincze

**Affiliations:** Department of Molecular Biology & Genetics, Faculty of Science & Technology, Aarhus University, Slagelse, Denmark; Department of Agriculture and Food Western Australia, Australia

## Abstract

Zinc deficiency is causing malnutrition for nearly one third of world populations. It is especially relevant in cereal-based diets in which low amounts of mineral and protein are present. In biological systems, Zn is mainly associated with protein. Cereal grains contain the highest Zn concentration during early developmental stage. Although hordeins are the major storage proteins in the mature barley grain and suggested to be involved in Zn binding, very little information is available regarding the Zn fertilization effects of hordein transcripts at early developmental stage and possible incorporation of Zn with hordein protein of matured grain. Zinc fertilization experiments were conducted in a greenhouse with barley cv. Golden Promise. Zn concentration of the matured grain was measured and the results showed that the increasing Zn fertilization increased grain Zn concentration. Quantitative real time PCR showed increased level of total hordein transcripts upon increasing level of Zn fertilization at 10 days after pollination. Among the hordein transcripts the amount of B-hordeins was highly correlated with the Zn concentration of matured grain. In addition, protein content of the matured grain was analysed and a positive linear relationship was found between the percentage of B-hordein and total grain Zn concentration while C-hordein level decreased. Zn sensing dithizone assay was applied to localize Zn in the matured grain. The Zn distribution was not limited to the embryo and aleurone layer but was also present in the outer part of the endosperm (sub-aleurone layers) which known to be rich in proteins including B-hordeins. Increased Zn fertilization enriched Zn even in the endosperm. Therefore, the increased amount of B-hordein and decreased C-hordein content suggested that B-hordein upregulation or difference between B and C hordein could be one of the key factors for Zn biofortification of cereal grains due to the Zn fertilization.

## Introduction

Zinc (Zn) is an essential element for plants and animals. After iron, Zn is the most abundant transition metal in organisms and is also present in all six enzyme classes [1]. Zinc deficiency is considered one of the top priority micronutrient deficiency problems affecting nearly one third of the world population [2]–[4]. In biological systems Zn is known to be incorporated with protein and prefers tetrahedral coordination by four ligands such as sulphur from cysteine, nitrogen from histidine, oxygen from aspartate and glutamate; much more rarely observed ligands include the hydroxyl of tyrosine, the carbonyl oxygen of the protein backbone and the carbonyl oxygen of either asparagine or glutamine [5], [6]. Bioinformatics searches for known zinc binding motifs identified that the human proteome contains 10–15% zinc binding proteins; and in *Arabidopsis* a total of 2367 proteins in 181 gene families are identified as Zn-related [1], [7], [8]. However, these figures do not reflect the total number of actual zinc binding proteins which might exceed these numbers since a lot of zinc binding motifs are impossible to predict with bioinformatics analyses [9], [10]. In addition to undiscovered potential zinc binding motifs, there are intermolecular binding sites (in which Zn ion acts as a bridging ligand between two polypeptides) in the sequences that are extremely difficult to predict in silico [7], [11].

Although cereal grains inherently contain lower amounts of proteins and minerals than some legumes [1], [12] up to 75% of the daily calorie intake of people living in the rural areas of the developing world comes from cereal-based foods with very low Zn bioavailability and concentrations (www.harvestplus.org).

The cereal grains have several major depositories for nutrients such as testa or pericarp, embryo including the scutellum and endosperm, surrounded by the aleurone layer. Usually the inner part of the endosperm has the lowest concentration of Zn and proteins and higher concentration of starch. The embryo and aleurone layers contain about half of the total Zn of cereal grains but during the milling process they are mostly removed [13]. Therefore, in order to improve the problem of Zn malnutrition, zinc concentration inside the endosperm needs to be increased.

A link among Zn transport and Zn storage proteins as well as nitrogen, sulphur and various amino acids was suggested from a nitrogen fertilization experiment [14]. In wheat, 0.26% increase of grain protein concentration is found with every mg of Zn per kg of top soil [15]. A study of bread wheat (*Triticum aestivum*) with ^65^Zn application at anthesis shows that the greatest proportion of the ^65^Zn is found in the glutenin fraction suggesting that Zn is associated with the grain storage proteins [16]. Furthermore, a strong correlation between wheat gliadin and grain Zn concentration was observed and suggesting that grain Zn in wheat could be bound to sulphur-rich low molecular weight (30–50 kDa) prolamins (γ and α gliadin; and B and C type LMW glutenins) [17]. Increasing Zn application increases the total polymeric glutenin compared to monomeric gliadin but within the glutenin fraction SDS-unextractable large polymeric glutenin is decreased compared to LMW glutenin [18]. It is also suggested that Zn could be primarily bound to protein/peptides in barley grain [19]. Therefore, these facts gave an indication that probably some fraction(s) in barley prolamin that is homologous to γ and α gliadin and LMW glutenin of wheat are capable of binding zinc.

In addition, it is known that Zn has a high affinity to the cysteine groups in disulphide bridges [20] and could be involved in the polymerization processes, which occur late in the grain filling [21], [22]. In wheat more than 80% of the total protein fraction (w/w) in the mature endosperm is gluten protein; either as monomeric (gliadins) or polymeric (glutenins) [21], [23]. The glutenin polymers are stabilized by inter- and intra-chain disulphide bridges and hydrogen bonds, and a positive relationship between dough strength and the ability of gluten proteins to form glutenin polymers has been observed [21], [24]–[27]. Moreover, increasing of Zn concentration in the grain by foliar Zn fertilization in bread wheat altered the gluten protein composition of the endosperm in favour of the polymeric glutenin. This gave an indication that a high glutenin to gliadin ratio is a trait connected to a high Zn concentration, [18].

In rice (*Oryza sativa*) it was observed that the distribution of Zn changed rapidly during grain development [28]. For instance at 10 days after fertilization (DAF), Zn is abundant in the aleurone layer, thereafter Zn decreases around the aleurone layer and spreads into the inner endosperm adjacent to the aleurone layer; *i*.*e*., the sub-aleurone layer [28]. A study using high-definition synchrotron X-ray fluorescence and ICP-MS in matured barley grain showed that in total 58% of zinc ion is present across the testa-aleurone-endosperm gradient [29]. Pearling and immunocytochemical studies of barley also have shown that protein-rich sub-aleurone cells are enriched in B-hordein (S-rich) and C-hordein (S-poor) [27] while D-hordein (the HMW prolamin) is only present in significant amounts in the inner part of the starchy endosperm [22], [23], [30]. Therefore, it could be assumed that major Zn binding storage proteins in the sub-aleurone layer of barley and wheat belong to proteins homologous to LMW-GS, possibly B-hordeins.

In wheat, the highest Zn concentration in the grain was found at the beginning of grain development such as 10–12 day after pollination (DAP) [31], [32]. Therefore, it gives an indication that endosperm proteins expressing in the early developmental stages could be potential sinks of Zn ion, especially those localized in the sub-aleurone layer. A transcriptomics study in barley showed that the proportion of B-hordein among all hordeins are higher at early developmental stages such as 10 DAP [33].

Considering the importance of barley as an ancient cereal grain crop ranking fourth among all crops in dry matter production in the world [34], the aims of the work were (i) to assess the effect of foliar and soil Zn fertilization on hordein transcript of early developmental stages; (ii) to seek a correlation between the proportion of hordein proteins and Zn concentration of the matured grain; and (iii) to detect the hordein fractions with Zn ion binding capabilities.

## Materials and Methods

### Plant material

Barley (*Hordeum vulgare* cv. Golden Promise) grains were surface sterilized by soaking in 30% H_2_O_2_ for 10 min, rinsed with distilled water 5 times; 3 grains were planted in a pot containing 200 g soil and grown under greenhouse conditions under a cycle of 16 h illumination and 8 h darkness at 23 and 18°C, respectively, at Research Centre Flakkebjerg, Slagelse, Denmark. After sowing, the pots were watered three times a week. Once germination was completed, plants were thinned to one plant per pot.

Individual spikes were tagged at flowering and harvested in the morning (09.00–11.30) at 10 days after pollination (DAP). The collected spikes were immediately frozen in liquid nitrogen and stored at −80°C until the analysis. The remaining spikes were harvested at maturity.

### Soil preparation and fertilizer application

Two hundred g dried PindstrupUnimuld (PindstrupMosebrug A/S, Denmark) soil was put in each plastic pot (size 1 L) without holes. Soil fertilizer was applied during soil preparation by adding 1 L fertilizer solution gradually into the soil until it was absorbed. The fertilization was repeated at 21, 45, 65 and 90 days after sowing the seeds. Foliar application (4 mL for each plant) was done with a hand sprayer twice in a week from 35 to 90 days after sowing the seeds. Three different Zn treatments were applied in the experiment: low, medium and high. In the low Zn treatment fertilizer solution [35], [36] containing basic nutrient (composition in mg: (NH_4_)_2_SO_4_ 48.2, MgSO_4_ 65.9, K_2_SO_4_ 15.9, KNO_3_ 18.5, Ca(NO_3_)_2_ 59.9, KH_2_PO_4_ 24.8, C_6_H_5_FeO_7_ 5.0, MnCl_2_·4H_2_O 0.9, CuSO_4_·5H_2_O 0.04, H_3_BO_3_ 2.9, H_2_MoO_4_ 0.01) was added into the soil of each pot and the leaves were sprayed with water. The medium and high Zn treatments were done by adding of basic nutrient solution supplemented with 0.25 mM and 1 mM of ZnSO_4_.7H_2_O into the soil respectively, plus foliar spraying of ZnSO_4_.7H_2_O solution on each plant: 1 mM for medium and 10 mM for high treatments.

### DNA and RNA extractions, mRNA isolation, cDNA synthesis

DNA coding actin gene (HVSMEi0002G07f) was prepared from plasmid clones using GenElute Plasmid Miniprep kit (Sigma-Aldrich) and DNA quantification was done using HoeferDyNA Quant 200 fluorometer and quantification assay (Sigma-Aldrich) according to manufacturer’s protocol.

Total RNA was extracted from two biological samples per treatment representing 2–3 grains taken from the middle part of the spike from each individual plant from different pot. The grains were homogenized in liquid nitrogen using a mortar and pestle, with either RNeasy kit (Qiagen) or TRI Reagent (Sigma-Aldrich) according to the manufacturers’ protocols. RNA qualities were checked using an Agilent 2001 Bioanalyzer (Agilent Technologies, Inc.) and mRNA was isolated from total RNA with Dynabeads (Invitrogen) according to manufacturer’s protocol. First strand cDNA was synthesized with 10 µL of mRNA, 1 µL of oligodT (Invitrogen), 4 µL of 5Xbuffer (supplied with enzyme), 1 µL RNAsin (Promega), 2 µL of 0.1 M DTT (supplied with enzyme), 1 µL of each dNTP (10 mM each) and 1 µL of Superscript II RT enzyme. The resulting cDNA mixture was diluted to 200 µL by adding 180 µL of MilliQ-water and stored at −20°C.

### Quantitative real time PCR (qRT-PCR)

Quantitative RT-PCR was performed in triplicate from each biological samples in 384 well microtiter plates in 7900HT Sequence Detection System (Applied Biosystems). The total reaction volume was 10 µL which contained 1 µL of appropriately diluted template DNA, 2.8 µL of MilliQ water, 0.6 µL of each primer (5 µM) and 5 µL of Power SYBR Green Master Mix (Applied Biosystems). Additional no-template control (NTC) reactions were carried out to check the potential of primer-dimers formation. The thermal profile set up, data analyses and quantification of individual gene of interest using actin standard and reference gene was done according to Kaczmarczyk et al. [33]. Our study conforms to the Minimum Information for Publication of Quantitative Real-Time PCR Experiments (MIQE) [37].

Statistical distributions and interpreting P values were done using online GraphPad software (http://www.graphpad.com/quickcalcs/distMenu/).

### Dithizone staining of matured grains

In order to study the localization of Zn in the matured grains, a staining method was developed using dithizone (DTZ), which creates a red/purple Zn-dithizonate complex upon binding with Zn [31], [38], [39]. Matured barley grains were incubated in milli-Q water for 1 hour and thereafter cut into two pieces longitudinally by scalpel/blade. The half grains were incubated in incubation buffer containing 100 mM Tris-HCl (pH 6.8), 50 mM NaCl and 10 mM DTT for 2 hours followed by half hour incubation with 50% methanol in the incubation buffer but without DTT. After incubation, the grains were stained in 1 mM DTZ (diluted with DMSO from 10 mM DTZ stock solution made with pure acetone) for 30 min. The stained grains were rinsed with buffer containing 50 mM Tris-HCl, pH 7.0 and 50 mM NaCl for enhanced colour reaction, following rinse with water and analysed qualitatively by using a reflectance light microscope (Carl Zeiss Microsystem) with a high-resolution digital camera (AuxioCam MRc5).

### Determination of grain Zn concentration

Mature grain Zn concentration (µg/g) was measured from all the biological replicates (plant from individual pots). All of the grains from the individual plants were grinded using a ceramic grain mill (KoMoFidibus 21) and Zn contents were measured from the flour by inductively coupled plasma optical emission spectrometry (ICP-OES) at Department of Plant and Environmental Sciences, Faculty of Science, University of Copenhagen.

### Hordein extraction and SDS-PAGE

The milled harvested matured barley grains were used for hordein determination as well. Barley alcohol-soluble proteins (hordeins) were extracted from 50 mg of flour and the isolated proteins were separated on SDS-PAGE according to Uddin et al. [40]. Maltose binding protein (MBP5) (6 mg/mL stock) (New EnglandBioLabs) as a negative control and yeast alcohol dehydrogenase (1 µg/µL stock) (Sigma-Aldrich Inc) as a positive control was used with following dilutions in the sample buffer: maltose binding protein (MBP5) in 1∶20; and alcohol dehydrogenase in 1∶1. Pre-stained high molecular weight protein standard (HiMark) were purchased from Invitrogen (Life Technologies).

### Coomassie staining and calculating the percentage of the band volume

After the SDS-PAGE gel was stained with Coomassie blue [41] and image was taken using BioRadGelDoc. ImageLab 4.01 was used for image analysis and calculating percentage of band volume in each lane. Different hordein bands were assigned according to their approximate molecular weight. Since B- and γ-hordein have similar molecular weight and with 1-D gel, it was not possible to distinguish them, in calculation they were assigned together. The ratio among different hordeins (from the band intensity) was calculated manually in MS excel 2007. Statistical distributions and interpreting P values was done using online GraphPad software (http://www.graphpad.com/quickcalcs/distMenu/).

### Blotting, radioactive ^65^Zn assay

Hordeins extracted from mature barley grain, separated by SDS-PAGE were blotted on membrane, renatured, overlayed and probed with zinc and subsequently zinc binding specificity of certain proteins was detected by autoradiography as described by Uddin et al. [40].

### Bioinformatics analyses

Primers for hordein gene families were used according to Kaczmarczyk et al. [33] For multiple sequence alignment of proteins, sequences were collected from uniprotKB (http://www.uniprot.org/help/uniprotkb) database and alignment was done using MEGA5.1 software with clustalW [42] algorithm using the following parameters: Gap penalty = 10; gap extension penalty = 0.2; Protein weight matrix = Gonnet, residues specific penalty = on, hydrophobic penalty = on, gap separation distance = 4, End gap separation off, and delay divergent cut off = 30%.

## Results

### Effects of Zn fertilization on the Zn concentration of the matured grain

The soil used in our experiments has very little amount of Zn (0.4 g/m^3^) and the low Zn treated plants (no additional Zn was applied during the whole growing period) received only slight amount of Zn from soil. We used ZnSO_4_.7H_2_O as a source of Zn fertilizer in the medium and high Zn treatments. ZnSO_4_ is the most widely applied fertilizer by the farmer due to high solubility, low cost and availability in the market. Moreover, it is also recommended that in case of biofortification of staple food crops fertilizers with high water-soluble Zn would be the best choice for foliar applications (http://www.zinc.org/crops/resources/publications).

In our experiments, foliar and soil Zn fertilization resulted in an increase of grain Zn concentration. Average matured grain Zn concentration for different Zn treated plants was measured as 65, 151 and 466 µg/g for low, medium and high Zn treatment respectively (Table 1). In comparison to low Zn treated plants the Zn concentration was increased 2.3 and 7.1 folds for the medium and high Zn treated plants respectively (Table 1). Our results are in agreement with previous reports, suggesting that foliar ZnSO_4_ application together with soil Zn fertilization is an effective way to promote grain Zn concentration in rice and wheat [31], [43], [44].

**Table 1:** Biological replicates and Zn concentration of full grain flour from matured barley (*Hordeum vulgare* cv. Golden Promise) measured (by ICP-OES).

Low Zntreatedplants*	Grain Znconcentration(µg/g)	Medium Zntreatedplants*	Grain Znconcentration(µg/g)	High Zntreatedplants*	Grain Znconcentration(µg/g)
P13	56.7	Q11	114.6	R15	239.7
P2	58.2	Q13	119.8	R11	349.2
P7	59.9	Q12	130.5	R14	391.1
P6	64.4	Q9	135.5	R2	395.3
P4	64.5	Q10	147.0	R1	401.2
P11	86.1	Q4	149.7	R6	433.4
		Q5	157.6	R8	525.8
		Q2	165.7	R13	597.2
		Q1	239.6	R7	650.6
				R4	680.7
Average	65.0	Average	151.1	Average	466.4
St. Error	4.4	St. Error	12.4	St. Error	44.9

* P2, P4, P6, P7, P11, P13; Q1, Q2, Q4, Q5, Q9, Q10, Q11, Q12, Q13; and R1, R2, R4, R6, R7, R8, R11, R13, R14, R15 are referring to each biological replicate (one plant in each pot).

### Effects of Zn treatment on hordein transcript at 10 DAP

**Figure 1:**
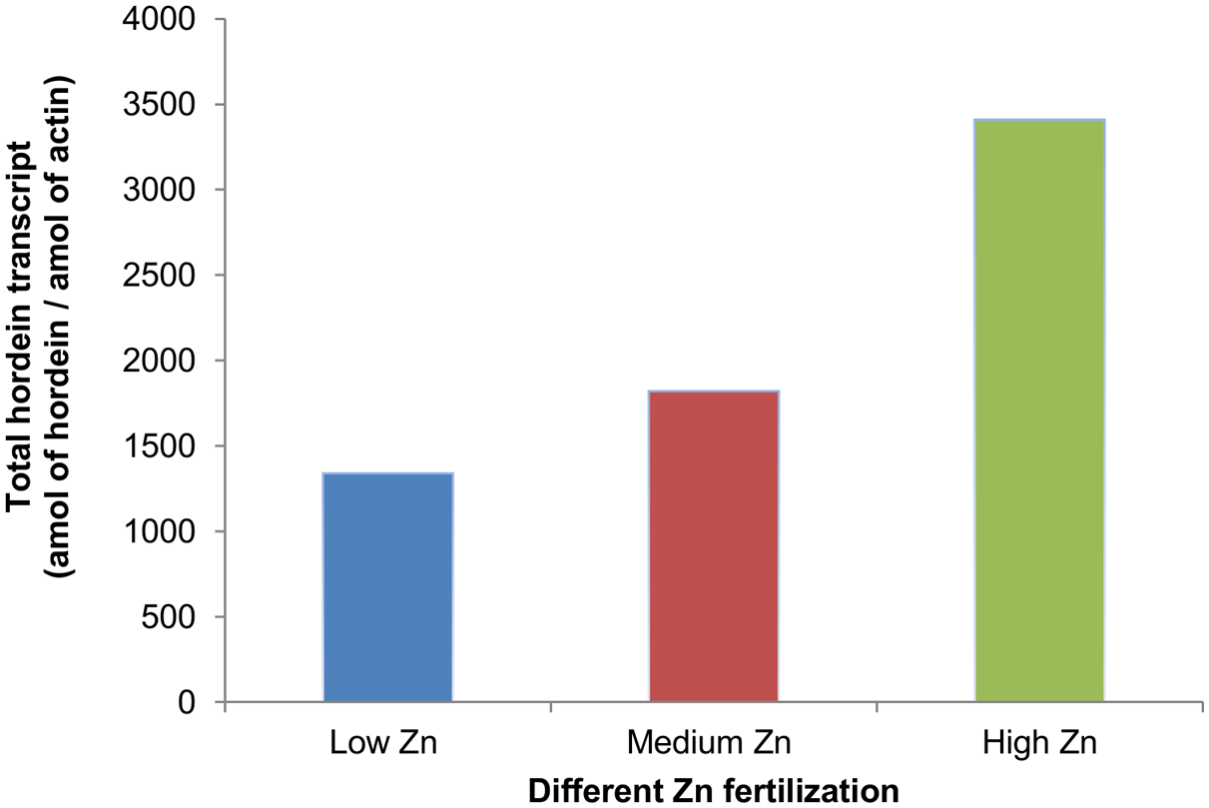
Effects of Zn fertilizations (low - blue; medium - red; high – green) on total amount of hordein transcripts (the sum of all hordeins) measured in amol of hordein/amol of actin.

Zn fertilization increased the total amount of hordein transcripts (measured in amole of hordein/amole of actin) (Figure 1). The observed increases compared to the low Zn treatment were 1.3 and 2.5 fold for the medium and high zinc treated group respectively (Figure 1).

**Figure 2:**
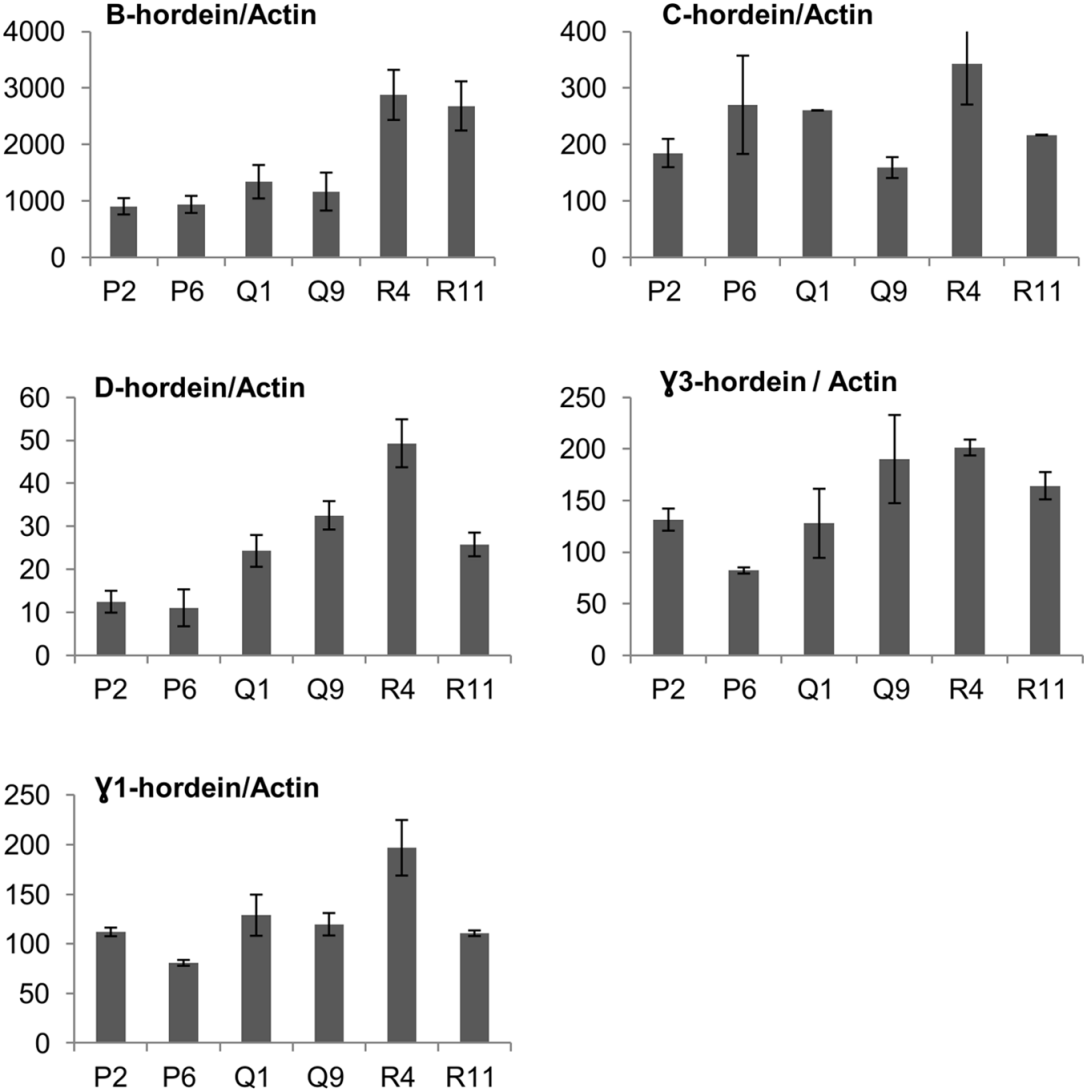
Effects of Zn treatments on the relative expression of different hordein transcripts at 10 DAP measured in amol of hordein/amol of actin. Two biological replicates for each Zn treatment are shown as: Low (P2 & P6), Medium (Q1 & Q9) and High (R4 & R11); and 3 technical replicates presented as means ± SE.

In addition, Zn concentration of matured grain also correlated with some of the hordein groups expressed at the early developmental stages (10 DAP). Transcripts of B-hordeins were found to be highly abundant followed by C-, γ- and D-hordeins in all three Zn treatments (Figure 2).

### B-hordeins

Using the primer sets designed to recognize the whole B-hordein gene family [33] we found that the steady state level of B-hordein transcripts increased with the increasing Zn concentrations (Figure 2). Average transcripts of B-hordein were measured as 922, 1254 and 2780 in amole of B-hordein/amole of actin for low, medium and high Zn fertilization/treatment respectively (Figure 2). Also a significant correlation (r = 0.915; DF = 4; the two-tailed P value = 0.0105) was observed between B-hordein expression at 10 DAP and matured grain Zn concentration (Table 2, Figure S1).

**Table 2:** Linear correlation between matured grain Zn concentration and hordein transcript at 10 DAP or protein from the matured grain.

Types ofhordeins	Correlationcoefficient (r)	Degrees offreedom (df)	Two tailedP-value
**Transcripts** *			
B	0.915	4	0.010
B1	0.891	4	0.017
B3	0.957	4	0.002
γ1	0.654	4	0.158
γ3	0.671	4	0.144
C	0.714	4	0.111
D	0.878	4	0.02
**Protein (%)** **			
(B+γ)	0.630	23	0.0007
C	0.401	23	0.0464
D	0.106	23	0.6130
(B+γ)–C	0.657	23	0.0004
(B+γ+D)–C	0.697	23	0.0001

* 10 DAP (amol of hordein/amol actin).

** % of protein from matured grain.

Similar trends were observed by using the primers sets [33] recognizing the two major sub-families of B-hordeins: B1 and B3. For instance, average transcript of B1-hordein was found as 262, 454 and 560 amole of B1-hordein/amole of actin for low, medium and high Zn fertilization/treatment respectively. Also a significant correlation (r = 0.89; DF = 4; the two-tailed P value = 0.017) was observed between B1-hordein expression at 10 DAP and matured grain Zn concentration (Table 2, Figure S1). For B3 hordein, average transcript was measured as 254, 402 and 594 amole of B3-hordein/amole of actin for low, medium and high Zn fertilization/treatment respectively. In addition, very significant correlation (r = 0.96; DF = 4; the two-tailed P value = 0.003) was found between B3-hordein expression at 10 DAP and matured grain Zn concentration (Table 2, Figure S1).

### C-hordeins

The C-hordein family (MW 55–75 kDa) is a S-poor prolamin group with unique sequences comprising highly conserved tandem repeats [45]. In matured barley grain C-hordeins account for 10–20% of the total hordein protein [25]. It was considered that C-hordein, like most of the S-poor prolamins, are lacking cysteine, and hence unable to form disulphide bonds [25].

In our Zn treatment experiments, on average very slight up-regulation of C-hordein transcript was observed with increasing the Zn concentrations (Figure 2) and average transcript was measured as 228, 246 and 280 amole of C-hordein/amole of actin for low, medium and high Zn fertilization/treatment respectively (Figure 2). Furthermore, no significant correlation (r = 0.71; DF = 4; the two-tailed P value = 0.11) was found between C-hordein expression at 10 DAP and matured grain Zn concentration (Table 2, Figure S1).

### D-hordeins

D-hordeins (MW>100 kDa), account for 2–4% of total grain protein in the mature grain and belong to HMW prolamins typified by the HMW subunits of wheat glutenin [25], [46].

In our experiment transcripts of D-hordein at 10 DAP were increased upon increasing Zn fertilization. The average transcript of D-hordein was measured as 12, 28 and 38 amole of D-hordein/amole of actin for low, medium and high Zn fertilization/treatment respectively (Figure 2). Furthermore, significant correlation (r = 0.88; DF = 4; the two-tailed P value = 0.021) was observed between D-hordein expression at 10 DAP and matured grain Zn concentration (Table 2, Figure S1).

### γ-hordeins

γ-hordeins belong to S-rich prolamin group (MW 36–44 KDa) and are represented by a small group of proteins, their contribution to the total grain protein has not been precisely determined [33], [47] although they are thought to be present in very minor amounts [48].

In our experiment, we used the common primers [33] designed for γ1- and γ3- subfamily. Towards higher Zn treatment average γ3-hordein was more responsive than the γ1 hordeins (Figure 2). The average γ3-hordein transcript level was measured as 107, 159 and 183 amole of γ3-hordein/amole of actin for low, medium and high Zn fertilization respectively while the average γ1-hordein transcripts was 96, 124 and 154 amole of γ3-hordein/amole of actin for low, medium and high Zn fertilization respectively (Figure 2). However, none of the γ-hordein showed statistically significant correlation between the expression at 10 DAP and matured grain Zn concentration [γ1 (r = 0.65; DF = 4; the two-tailed P value = 0.15); and γ3 (r = 0.67; DF = 4; the two-tailed P value = 0.14)] (Table 2, Figure S1).

### Effect of Zn fertilization on the ratio of different hordein transcripts

Prolamins consist of multiple gene protein families, which are divided into monomeric and polymeric groups. In barley, C-hordein and γ-hordeins are the monomeric prolamins and B- and D-hordeins are considered as polymeric hordeins [49]. In our experiment, although a slight increase was observed in the transcript level of monomeric hordeins at 10 DAP, this increase did not significantly correlate with actual matured grain Zn concentration (Table 2, Figure 2 & Figure S1).

**Figure 3:**
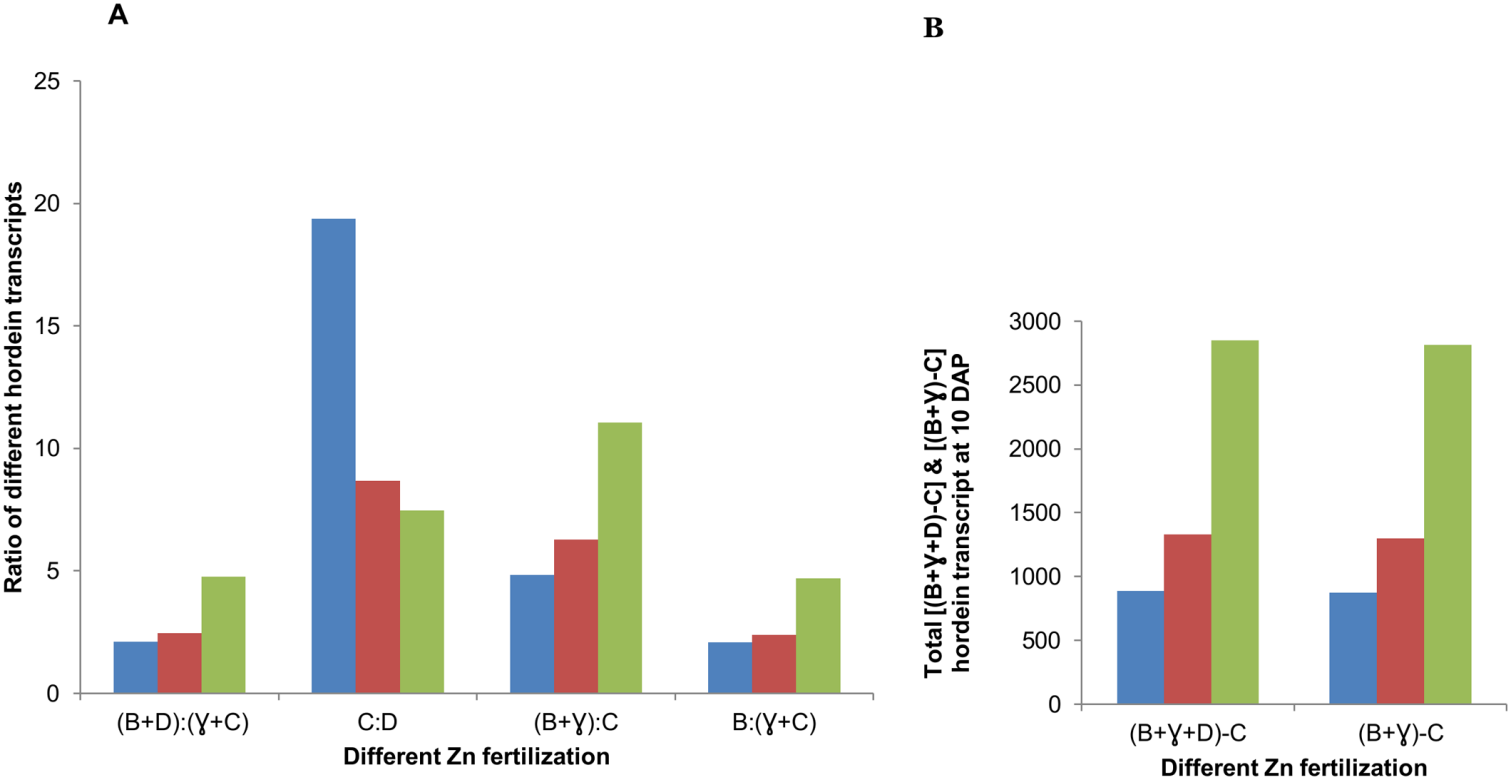
Effects of Zn treatments on ratio of different hordein transcripts measured at 10 DAP. Zn treatment labels: low - blue; medium - red; high – green. A) Ratio of different hordein transcripts measured at 10 DAP from different Zn fertilization; B) Total [(B+γ+D)–C] hordein or [(B+γ)−C] hordein transcripts measured at 10 DAP from different Zn fertilization. In the figure B, C, D and γ refers as B-hordein, C-hordeins, D-hordeins, and γ-hordeins respectively.

In contrast to monomeric hordeins, polymeric hordeins such as B- and D-hordeins were up regulated at 10 DAP with increasing Zn fertilization and this up regulation also correlated with the actual Zn concentration in matured grain (Table 2, Figure 2 & Figure S1). B-hordeins are S-rich and are the main group of hordeins in barley (70–80% of total prolamin content), and as sub units they are about the same size as γ-hordein [49]. D-hordeins are similar to HMW subunit of wheat [50]. In our experiment, the ratio in the expression data from different hordein transcripts showed an increase of glutenin and gliadin ratio [(B+D):(C+γ)] and the ratio between LMW glutenin and gliadin sub unit [B: (C+γ)] with increasing Zn treatment, whereas ratio between monomeric gliadin and polymeric HMW glutenin (C:D) decreased upon higher zinc treatment (Table 2, Figure 3). In addition, increasing Zn fertilization also increased the percentage of [(B+γ)−C] -hordeins or [(B+γ+D)–C]-hordein (Figure 3).

### Effects of Zn fertilization on hordeins of matured grain

**Figure 4:**
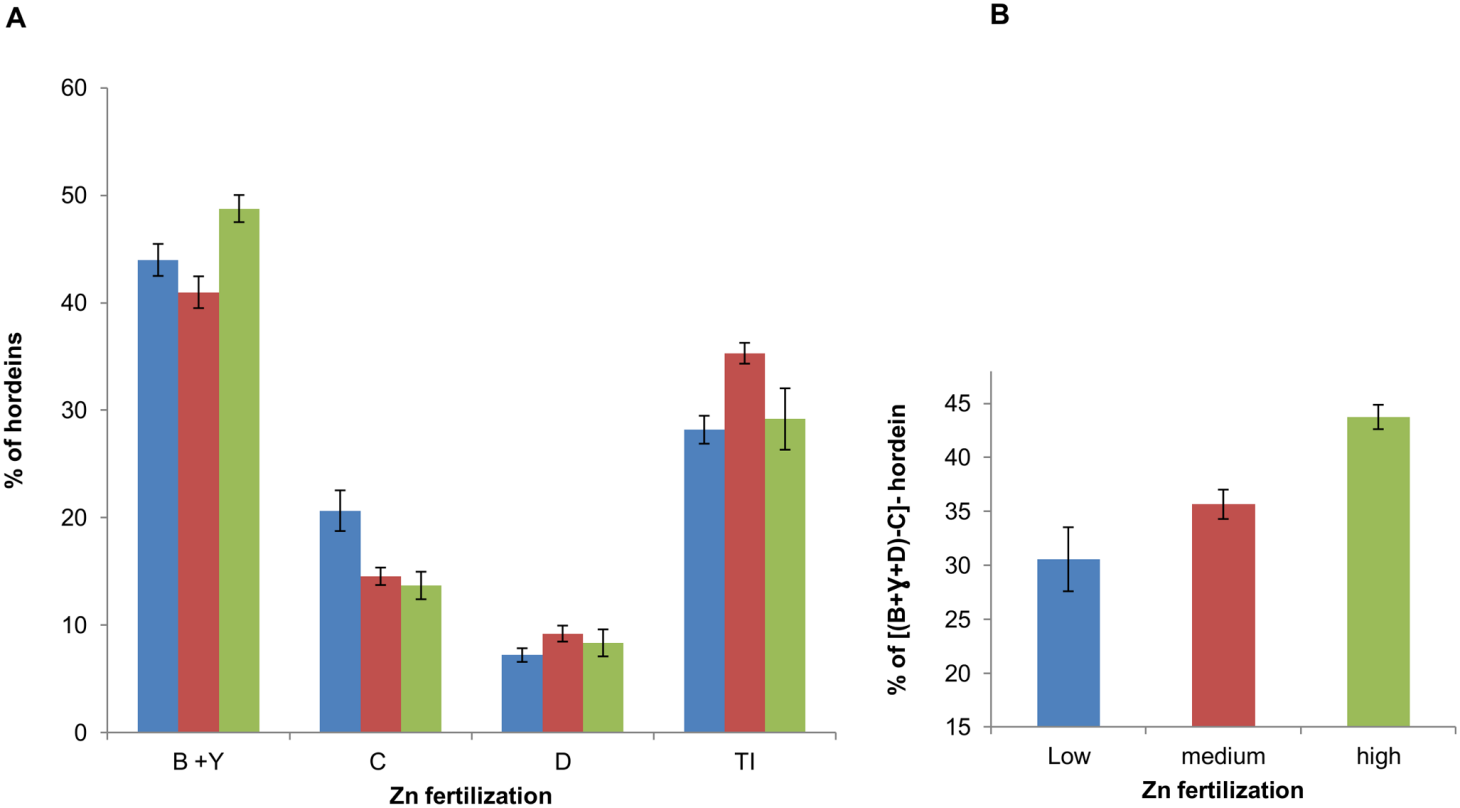
Effects of Zn treatments on percentage of different hordeins of matured grain measured from image analyses of SDS-PAGE gel. In the figure B, C, D, γ and TI refers as B-hordein, C-hordeins, D-hordeins, γ- hordeins and A-hordeins/Trypsin inhibitors/alpha amylase inhibitors respectively. The calculations are based on the biological replicates mentioned in table 1 and presented as means ± SE. A) Percentage of different hordeins after different Zn fertilization; Zn treatment labels: low - blue; medium - red; high - green. B) % of [(B+γ+D)–C]-hordein after different Zn fertilization.

The hordeins were isolated from all the biological replicates with known zinc concentration (Table 1) from matured grain and the different hordeins were separated on Coomassie stained SDS-PAGE gel. Image analyses of these protein gels demonstrated that on average the highest Zn treatment decreased the monomeric C-hordein whereas polymeric B- together with γ- and D-hordein was increased (Figure 4). However, plants from medium Zn treated groups showed a slight decrease of B-hordein whereas D-hordein as well as LMW S-rich prolamins such as trypsin/α-amylase inhibitors (known as A- hordeins previously, shown as TI in Figure 4A) was increased (Figure 4A). Although γ- and B-hordein have similar molecular weight we assume that this change of hordein percentages was due to the B-hordein since the amount of γ-hordein is very low in barley [51]. In addition, increasing Zn treatment also increased the differences between (B+γ+D) and C-hordein [% of (B+γ+D)–C] (Figure 4B).

Furthermore, very significant linear positive correlation (r = 0.63; DF = 23; the two-tailed P value = 0.0007) was observed between (B+γ)-hordein of matured grain and actual grain Zn concentration (Table 2), whereas negative correlation was found in case of C-hordein (Figure S2, Table 2). In contrast to the results from qPCR at 10 DAP, D-hordein did not show any significant correlation between matured grain Zn concentration and percentage of D-hordein present in matured grain (Table 2, Figure S1 & Figure S2).

In addition, increasing polymeric hordein also increased grain zinc concentration and highly significant positive correlation was found between grain Zn concentration and percentage of [(B+γ)−C] or [(B+γ+D)–C] hordeins in the grain (Table 2, Figure S2).

### Zinc localization of matured grain with dithizone staining

**Figure 5:**
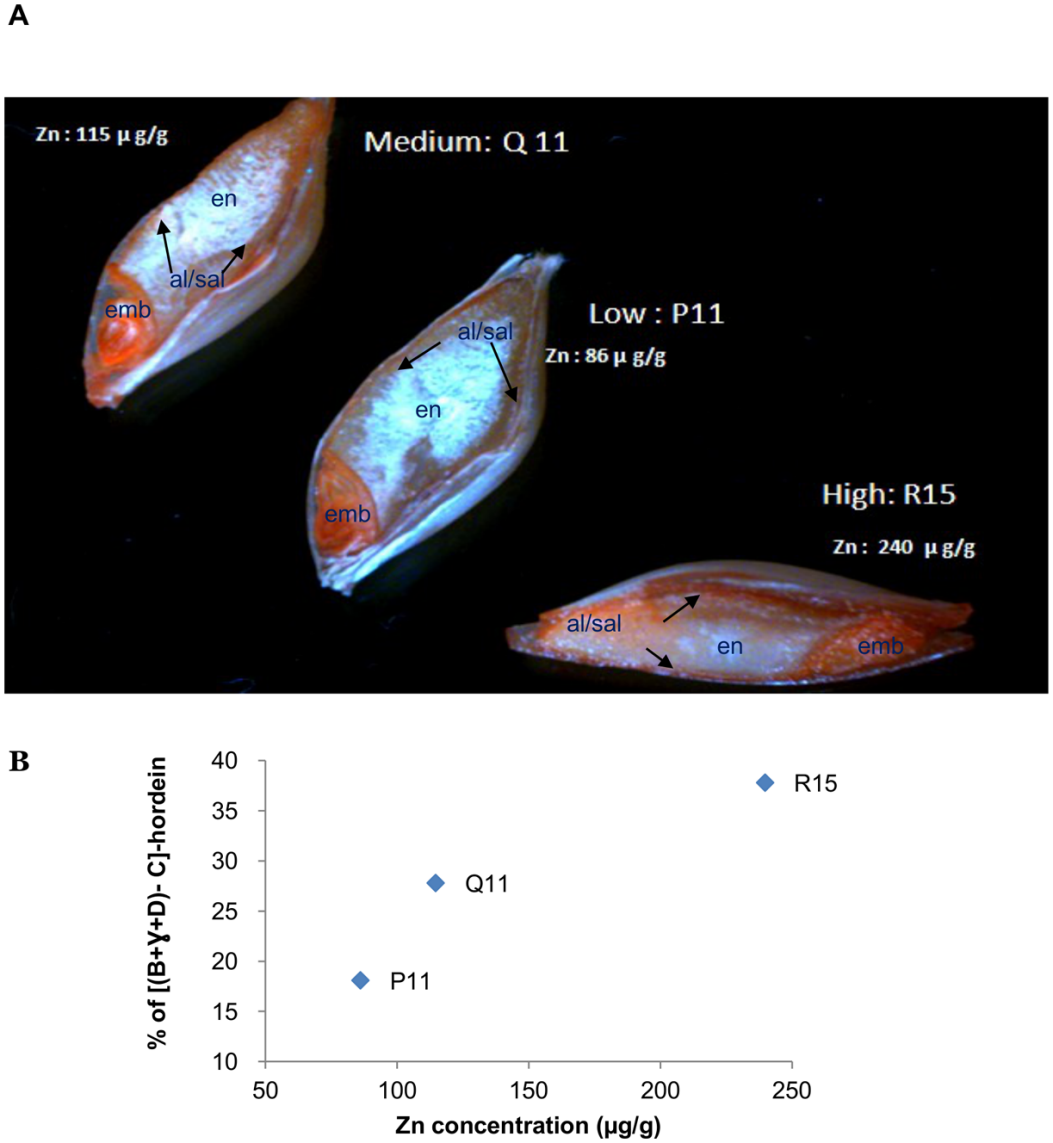
Zinc localization in matured grain. A) Dithizone staining of the mature grain from three different Zn fertilization experiments. Dithizone formed red/pink coloured complexes upon binding Zn ion. En- inner endosperm; emb- embryo surrounding region; al/sal - aleurone and sub-aleurone layers. B) Percentage of [(B+γ+D)–C]-hordein in the mature grains from three different Zn fertilization experiments. P11, Q11 & R15 refers as individual grains from low, medium and high Zn fertilization group.

Dithizone staining was performed for the localization of Zn inside the grain from different Zn treated plants. Regardless of the Zn treatment, embryo and aleurone/sub-aleurone layers showed the highest Zn intensity (Figure 5). However, high zinc application made a slight enrichment of Zn ion inside the endosperm in comparison to low zinc treated groups (Figure 5). This Zn enrichment inside the endosperm shown by DTZ staining was observed up to a certain limit (average total grain Zn concentration 240 µg/g), and beyond this limit colour intensity did not increase further (data not shown).

### Zinc blotting and detection of Zinc binding protein

In the ^65^Zn assay positive Zn binding was observed for the well-known positive control alcohol dehydrogenase (MW 38 kDa) and the protein bands (MW 35–46 kDa) from our hordein isolates, whereas maltose binding proteins (MBP5) (MW 42.5 kDa) used as negative control did not show any zinc binding (Figure S3). The molecular weights of hordein extracts showing positive bands in ^65^Zn autoradiography are about 35–46 kDa representing either as B-hordein or γ-hordein [25], [40].

### Multiple sequence alignment with Zn binding pumpkin trypsin Inhibitor

From crystallographic studies it was observed that pumpkin trypsin inhibitor, CMTI-I (ITR1_CUCMA) is capable of binding zinc ion in a tetrahedral and symmetric fashion through glutamic acid residue [52]. Like CMTI-I protein, B-hordeins have a trypsin/alpha amylase inhibitor domain in the C-terminal region which might participate in Zn binding [52], [53].

**Figure 6:**
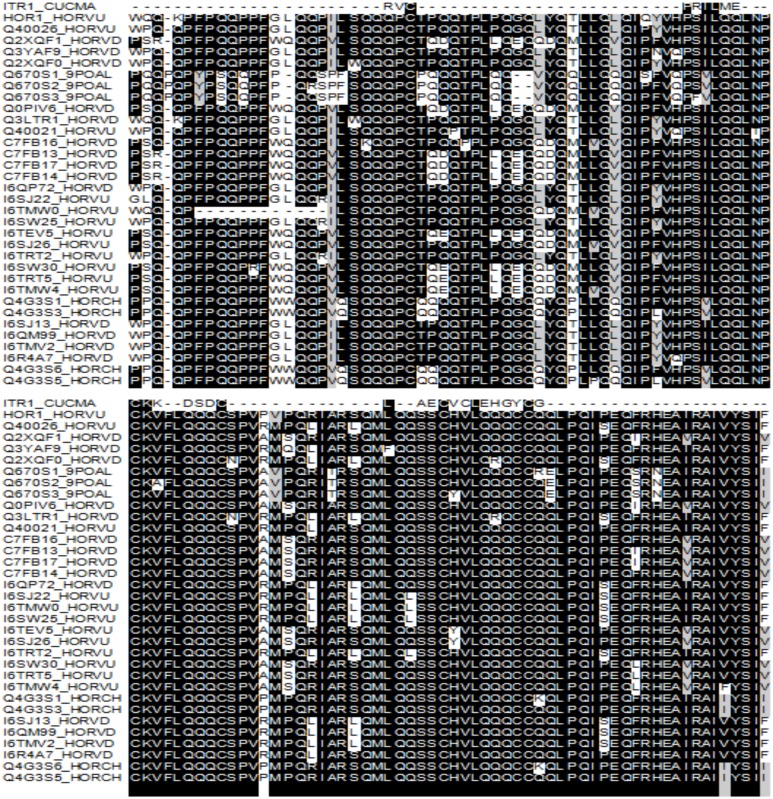
Multiple sequence alignment of zinc binding trypsin inhibitor 1 of *Cucurbita maxima* (ITR1_CUCMA) and several B hordein of *Hordeum vulgare*. The alignment was done using MEGA5.1 software with clustalW algorithm. Identical amino acids (conserved sites) are shaded in black.

Therefore, multiple sequence alignment was done with the full length amino acid sequence from pumpkin trypsin inhibitor (CMTI-I) and different B-hordein protein sequences (Figure 6). The multiple sequence alignment showed presence of 11 conserved sites in C-terminal region of B-hordein and CMTI-I protein, consists of cysteine (C), proline (P), isoleucine (I), leucine (L) and lysine (K) residues (Figure 6).

Zinc binding sites in protein are often distorted tetrahedral or trigonal bipyramidal geometry [6]. Histidine (H), cysteine (C), glutamic acid/glutamate (E/Q) and aspartic acid (D) are the most common amino acids that supply ligands to these sites. Usually in protein zinc-binding sites, zinc ion is coordinated by different combinations of protein side chains: the nitrogen from histidine, oxygen from aspartate or glutamate, and sulphur from cysteine; and among them histidine and cysteine are the most commonly observed [6], [54]. However, much more rarely found ligands are the hydroxyl of tyrosine, the carbonyl oxygen of protein backbone and the carbonyl oxygen of either asparagine or glutamine [6]. In this alignment, five cysteine residues were found as conserved in B-hordein and CMTI-I (Figure 6). Usually during protein evolution polar amino acids such as asparagine (N), aspartic acid (D), glutamic acid (E), glutamine (Q), serine (S), tyrosine (Y) can be substituted by each other [55]. For instance, the GLU19 residue of CMTI-I, which was found to bind zinc in a crystallographic study [52], was substituted by serine in B-hordein (Figure 6). Furthermore, glutamine (Q) is a very abundant amino acid in B-hordein [49] which can be post translationally modified by deamination and changed to glutamic acid (E) [48], [56] that might participate Zn binding [6].

## Discussions

### Zinc treatment increased the level of hordeins

Zinc exists in soil in various organic and inorganic forms, which affect its bioavailability to plants. More than 90% of zinc in the soil is insoluble and thus unavailable for plants [1]. Therefore, it is very important to keep sufficient amount of available Zn in soil (by soil Zn applications) and in leaf tissue (by foliar Zn applications) to maintenance of adequate root Zn uptake and transport of Zn from leaf tissue to the seeds during reproductive growth stage [57], [58]. The main goals of our experiments were to increase grain Zn ion concentration, especially in the endosperm and to explore the effects of zinc treatments on hordein protein composition of barley grain. Our experiments with combined foliar and soil Zn fertilization showed that by applying ZnSO_4_ the total hordein transcript levels were significantly increased with increasing Zn fertilization at 10 DAP (Figure 1). It is known that primary control of the synthesis of storage protein in wheat is at the level of transcription [59], [60]. Therefore, it is possible that increasing Zn fertilization increases DNA transcription, which results in an increase in all of hordein transcripts at 10 DAP. The major fraction of the hordein transcripts that showed up-regulations were B-hordeins (Figure 2).

In wheat, significant correlations have been observed between concentration of grain Zn and the protein content which showed that increasing supplies of Zn up to an optimum concentration could significantly increase the protein content of wheat grain [61], [62]. By examining data from 63 wheat crops in the Mallee region of South-eastern Australia over 3 growing seasons 0.26% increase in grain protein concentration is reported with every mg Zn per kg top soil [15]. Moreover, Zn speciation study by SEC-ICP-MS analyses of barley grain suggests that Zn is mainly binding with protein/peptide [19], [29] hence it is possible that increasing Zn concentration could increase total hordein protein or vice versa.

Considering the facts that hordeins are the major storage proteins in barley, our results showed that it was possible to increase matured grain Zn concentration and it was correlated with the increasing protein concentration of the barley hordeins. (Figure 1 & Figure S1).

### Association of Zn ion with B-hordein

In this experiment B- and D-hordein which are homologous to the glutenin subunit of wheat, showed significant correlation between grain zinc concentration and expression of transcript at 10 DAP (Figure S1). However, in the matured grain only the percentage of B-hordein showed significant positive correlation with grain Zn concentration, whereas C-hordein showed a negative correlation (Figure S2A). In wheat, labelling studies with ^65^Zn have shown that the glutenins have a much greater level of ^65^Zn incorporation (47–65%) than the other grain proteins [16]. Similar results were obtained from barley storage protein extracts and it is reported that western immunoblotting assay shows the presence of B-hordein within this MW range [40]. In addition, we assume that some protein bands in the lower molecular weight region (MW<20 kDa) which showed positive ^65^Zn binding probably belonged to trypsin/alpha amylase inhibitor families, previously known as A-hordein [40], [63] (Figure S3).

Therefore, our results are in agreement with the above mentioned studies that Zn can be incorporated in the glutenin fraction of grain, in this case B-hordein of barley, which is homologous to LMW-GS of wheat.

### B-hordein expression at early developmental stage and correlation with Zn concentration of matured grain

After foliar application of ZnSO_4_ the highest concentration of Zn ion in the grain of bread wheat is at the beginning of the grain development (10–12 DAP) and at later stages to maturity the relative concentration of Zn ion is stable or decreases [31]. In barley the proportion of the different hordein groups during grain development varies slightly but the amount of total hordein transcripts increased from 10 DAP onward and the B-hordein transcripts always appear as the highest proportion (more than 80%) compared to the other hordeins [33]. Zinc uptake rate of the wheat endosperm reaches its maximum level at 14 days after anthesis (DAA). During the most active starch deposition period (14 to 34 DAA) the demand for Zn from the endosperm cells remains stable or is slightly reduced [32]. The lack of Zn demand or low sink capacity of the starchy endosperm are the reasons for the low concentration of zinc in the endosperm, and therefore enhanced levels of Zn can only be reached when additional sink is created in the endosperm [32], [64]. Therefore, we assumed that B-hordein transcript at 10 DAP could not vary too much compared to the matured grain and the linear relationship between matured grain Zn concentration and proportion of B-hordein transcripts at 10 DAP could be also true for all stages of grain development. It could be suggested that B-hordeins are incorporating Zn and the percentage of B or [(B+γ+D)–C] is positively correlating with the concentration of Zn. Therefore, decreasing the percentage of C-hordein in the grain achieved by Zn fertilization could help to increase the total grain Zn concentration. Decreasing C-hordein could help to increase B-hordein content of the grain that might participate in Zn binding. Previously, a study with transgenic approach for down-regulation of C-hordein transcripts with RNAi antisense technique showed pleiotropic effects including up-regulation of B-hordein as well as other Zn binding proteins [65], [66]. The expression of B-hordein coding gene using D-hordein promoter could be another possible way to enrich the Zn concentration in inner endosperm. However, additional experiment with transgenic approach is needed to see whether increasing B-hordein could be another biofortification strategy.

### Zn could have a role in hordein polymerization

Usually monomeric prolamins contain intramolecular disulphide bonds, whereas polymeric prolamins have inter- and intramolecular disulphide bonds [48].

Foliar Zn ion application increases grain Zn concentration and has a special effect on protein composition by decreasing the proportion of gliadin in the flour, and increasing the ratio of polymeric protein to gliadin [18]. We also observed that increasing Zn application increased the ratio of polymeric to monomeric hordein transcript [(B+D):(γ+C)] (Figure 3A). In addition, transcript of total [(B+γ+D)–C] hordein or [(B+γ)−C] hordein also increased with the increasing Zn fertilization (Figure 3B). Image analyses of SDS-PAGE gel also showed an increase in percentages of [(B+γ+D)–C] hordeins (Figure 4B). In addition, our results showed significant linear correlation between percentage of [(B+γ+D)–C]- or [(B+γ)−C]- hordein and matured grain Zn concentration (Figure S2B) which is in agreement with the suggestion that sulfhydryl groups of the cysteine residue are involved in this polymerization [18]. Since Zn has a strong affinity to sulfhydryl groups and prevents their peroxidation to form disulphide bonds [20], the concentration of grain Zn could influence the degree of polymerisation of the proteins which could shift the balance to low molecular weight polymers (eg. B-hordein) [18]. In our experiment, this is consistent with the increase in B-hordein with increasing Zn concentration in the grain both at 10 DAP and at the matured stages.

### Zinc incorporation with B-hordein in sub-aleurone layer in endosperm

We reported that Zn concentration in matured grain was predominantly high in embryo, aleurone layer and outer part of the endosperm (sub-aleurone layers) (Figure 5A). However, increasing Zn fertilization could move/increase Zn concentration inside the endosperm up to a certain level and the difference of [(B+γ+D)–C]-hordein increased (Figure 5B). Our results are consistent with previous findings in developing wheat grain (subjected to foliar application of Zn) that Zn predominantly is located in the embryo, the aleurone layer and outer part of the endosperm (sub-aleurone layer) in the matured grain [31], [32], [58]. Zn distribution in barley grain is not limited to the aleurone layer but is also present in the outer part of the endosperm (sub-aleurone layers) which is known to be rich in proteins [29], [31]. In addition, Zn is present in the ventral part of the endosperm but found to be limited to the aleurone layer in the lateral and dorsal parts of the grain [67].

Pearling and immunocytochemical studies of barley have shown that the protein-rich sub-aleurone cells are enriched in B-hordein (S-rich) and C-hordein (S-poor) [27] while D-hordein (the HMW prolamin) is only present in a significant amount in the inner part of the starchy endosperm [68]. A study from scanning electron microscopy and mass spectrometry suggests that the structure of the aleurone layer storing large quantities of protein did not differ between the high- and low-protein forms of barley biotypes. However, the quantity of protein in barley is determined by proteins localized in the sub-aleurone layer [69]. Two proteins, B3-hordein and Z-type serpin present in the sub-aleurone layer make the biggest quantitative difference between high- and low-protein kernels in forms of barley. In our experiment, both B1- and B3-hordeins showed high responses towards Zn treatment at early developmental stage, and significant linear correlation was observed between B1- and B3-hordein transcript and grain Zn concentration of matured stage (Table 2). Therefore, our experiments showing that B-hordeins are capable of binding Zn are in agreement with those previous reports and it could be assumed that major Zn binding storage proteins in the sub-aleurone layer of barley and wheat belong to proteins homologous to LMW-GS.

### B-hordein expression at early developmental stage and correlation with Zn concentration of matured grain

Barley storage protein transcripts encoding hordeins (B, C, D & γ) start to appear in the endosperm/sub-aleurone fraction at the early developmental stage (about 10–12 DAP) and reach their highest levels from 16 DAP onward [70]. Experiments with foliar application of ZnSO_4_ in wheat showed highest concentration of Zn in the grain at the beginning stage (early milk) of the grain development (10–12 DAP) and at later stages to maturity Zn concentration was stable or decreased although the total amount of Zn increased due to the increasing dry mass volume of the grain [31]. Therefore, we choose to study hordein expression at the early stage of grain development (10 DAP). Kaczmarczyk et al. [33] studied expression of different hordein transcripts at different developmental stages and found that the proportion of the different hordein groups during grain developmental varied slightly but the amount of total hordein transcripts increased from 10 DAP onward and B-hordein always appeared as a higher proportion (more than 80%) than any other hordeins [33]. Therefore, from our experiment we assumed that the relative proportion of B-hordein transcript at 10 DAP did not vary too much compared to the matured grain and the linear relationship between matured grain Zn concentration and proportion of B-hordein transcripts at 10 DAP could be also true for all stages of grain development.

The Zn uptake rates of the wheat endosperm reaches its maximum level at 14 DAA and after the Zn concentration in endosperm remains more or less stable [32]. It is suggested that the low sink capacity of the starchy endosperm are the reasons for the low concentration of zinc in the endosperm, and therefore enhanced levels of Zn can be reached only when an additional sink is created [32], [64]. The sink capacity could be increased either by decreasing starch content or increasing the proportion of different hordeins that are incorporating Zn. B-hordeins could be that sink protein as we reported that the percentage of B or [(B+γ+D)–C] was correlating with the concentration of Zn. Therefore, a possible approach to enrich the Zn concentration in the inner endosperm could be the expression of B-hordein using D-hordein promoter to allow translocation in the endosperm. Alternatively decreasing C-hordein level could help to increase B-hordein content of the grain as it is shown in C-hordein antisense lines [65], [66]. We showed that increasing Zn fertilization could decrease percentage of C-hordein in matured grain (Figure 4), as well as the ratio of C/D transcript at the 10 DAP (Figure 3A). Therefore, decreasing the percentage of C-hordein in the grain could also help to increase the total grain Zn concentration.

### Hordein evolution and possible Zn binding sites/domain in B-hordein

Prolamins are thought to have evolved about 100 million years ago through gene duplication and addition/deletion of repetitive domains in the sequences of this ancestral protease inhibitor [49], [71]–[73]. B-hordein and CMTI-I, a small-protein trypsin inhibitor from pumpkin known to bind Zn ion, share a common trypsin inhibitor domain. Therefore, it is possible that one of several potential zinc binding sites/domains is located within this region of the B-hordein sequence (Figure 6). Usually zinc is considered as a “borderline” metal, which does not consistently act either “hard” (not very polarizable) or “soft” (highly polarizable); and hence does not have a strong preference for coordinating with either oxygen or nitrogen or sulphur atoms [5], [6]. Speciation of Zn in barley and wheat grain is not well understood although several hypotheses exist regarding the association with S or O atoms in the aleurone layers of the grain [19], [74]. B-hordein is known to be localized mainly in the sub-aleurone layer of the grain. Therefore, if S is the main ligand in the sub-aleurone layer of the grain then it is possible that B-hordein is participating in binding Zn through cysteine residues; Zn could either have a role in stabilizing the structure of the protein or be involved in the polymerization of hordein.

### Conclusions

Greenhouse experiments showed that combined foliar and soil Zn fertilization could significantly increase the total Zn concentration of barley grain. Although the total hordein transcripts increased in the grain at 10 DAP, the increase of B-hordein transcript level was the most prominent. Significant positive correlation was observed between the amount of B-hordein transcript level at 10 DAP and grain Zn concentration at the matured stage. Furthermore, increasing Zn fertilization also increased the percentage of B hordein and we found significant positive correlations between proportion of [(B+D)–C or (B–C)]-hordein and Zn concentration in the matured grain.

The zinc fertilization increased the total zinc concentration of the grain and the enrichment happened mostly around areas such as embryo, aleurone and sub-aleurone layer. The outer part of the endosperm (sub-aleurone layer) was enriched with Zn due to the increased presence of B-hordein. Considering the obtained Zn concentration of the grain and our protein data we concluded that not all hordein, but B-hordein upregulation or difference between B- and C-hordein is one of the key factors for increased Zn concentration in the mature grain. More experimental evidences are required to see whether down regulation of C-hordein level and up regulation of B-hordein could be a viable option for Zn biofortification.

## Supporting Information

Figure S1 Linear correlations between different steady state level of hordein gene at 10 DAP and Zn concentration of the matured grain.
(TIF)

Figure S2 Linear correlation between matured grain Zn concentration and different proportion of hordein measured from SDS-PAGE gel by image analyses. A) linear correlations of grain Zn ion concentration and % of hordeins; B) linear correlations of grain Zn concentration and percentage of [(B+γ+D)−C] or [(B+γ)−C]-hordeins.
(TIF)

Figure S3 Selective binding of zinc ion by alcohol soluble protein from barley (cv. Golden Promise) grain. A) Replica membrane stained with amido black, B) Zinc binding protein specified by autoradiography showing black bands on the membrane. In A & B: 1- Maltose binding protein (MBP5); 2- HiMark prestained protein marker, 3-Hordein extract; 4- Alcohol dehydrogenase. Numbers in vertical axis represent the approximate molecular weight (kDa) of the protein bands.
(TIF)
