# Supplementary figures and images for: Effects of Zn Fertilization on Hordein Transcripts at Early Developmental Stage of Barley Grain and Correlation with Increased Zn Concentration in the Mature Grain
Source: PLoS One. 2014 Sep 24;9(9):e108546. doi: 10.1371/journal.pone.0108546 (PMC4177403; doi:10.1371/journal.pone.0108546)

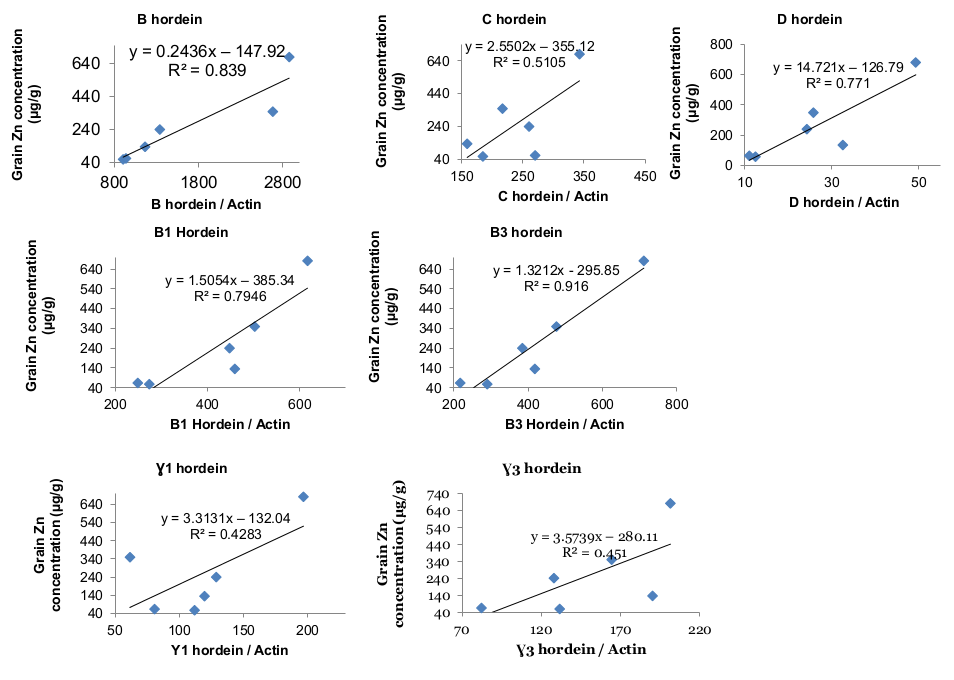

Supplement: Figure S1 — Linear correlations between different steady state level of hordein gene at 10 DAP and Zn concentration of the matured grain. (TIF) [file pone.0108546.s001.tif]

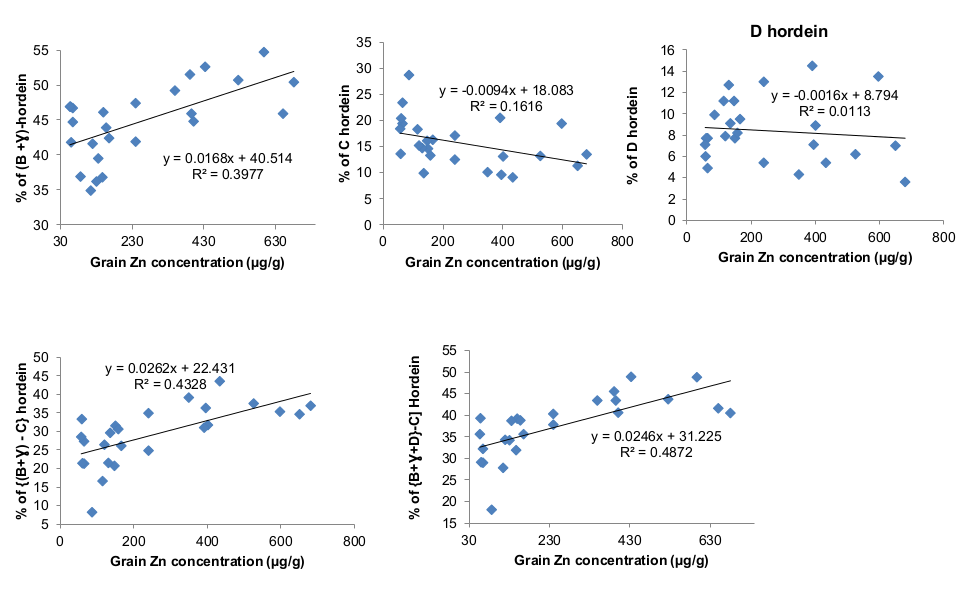

Supplement: Figure S2 — Linear correlation between matured grain Zn concentration and different proportion of hordein measured from SDS-PAGE gel by image analyses. A) linear correlations of grain Zn ion concentration and % of hordeins; B) linear correlations of grain Zn concentration and percentage of [(B+γ+D)−C] or [(B+γ)−C]-hordeins. (TIF) [file pone.0108546.s002.tif]

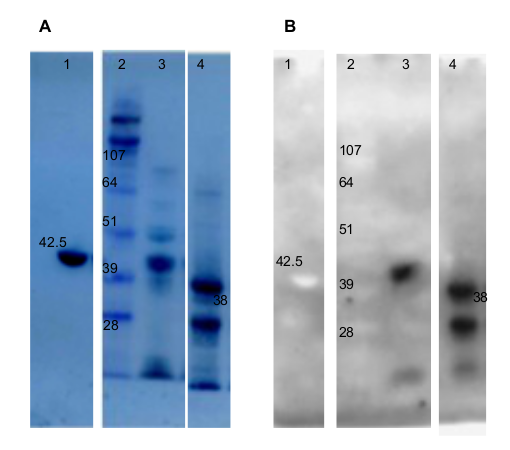

Supplement: Figure S3 — Selective binding of zinc ion by alcohol soluble protein from barley (cv. Golden Promise) grain. A) Replica membrane stained with amido black, B) Zinc binding protein specified by autoradiography showing black bands on the membrane. In A & B: 1- Maltose binding protein (MBP5); 2- HiMark prestained protein marker, 3-Hordein extract; 4- Alcohol dehydrogenase. Numbers in vertical axis represent the approximate molecular weight (kDa) of the protein bands. (TIF) [file pone.0108546.s003.tif]
